# Closing-in Behavior and Parietal Lobe Deficits: Three Single Cases Exhibiting Different Manifestations of the Same Behavior

**DOI:** 10.3389/fpsyg.2018.01617

**Published:** 2018-09-13

**Authors:** Elisabetta Ambron, Luca Piretti, Alberta Lunardelli, H. Branch Coslett

**Affiliations:** ^1^Laboratory for Cognition and Neural Stimulation, Neurology Department, Perelman School of Medicine, University of Pennsylvania, Philadelphia, PA, United States; ^2^Neuroscience Area, Scuola Internazionale Superiore di Studi Avanzati, Trieste, Italy; ^3^Azienda Sanitaria Università Integrata di Trieste, Trieste, Italy

**Keywords:** closing-in behavior, drawing, attention, executive functions, motor control

## Abstract

Closing-in behavior (CIB) is observed in copying tasks (graphic or gestural) when the copy is performed near or on the top of the model. This symptom has been classically considered to be a manifestation of constructional apraxia and is often associated with a visuospatial impairment. More recent work emphasizes the attentional and/or executive nature of the behavior and its association with frontal lobe dysfunction. We describe three patients in whom CIB was associated with posterior parietal deficits of different etiologies (stroke in Patient 1 and dementia in Patients 2 and 3). In copying figures, Patient 1 produced the shape with high accuracy but the rendering overlapped the model, while for Patients 2 and 3 the copies were distorted but overlapping or in close proximity to the target. In gesture imitation, Patient 2 performed the gestures toward the examiner’s space, while Patient 1 showed a peculiar form of CIB: when he was asked to place the ipsilesional arm in a position that mirrored the contralesional hand, Patient 1 moved his hand toward his contralesional hand. Patient 3 did not present gestural CIB. While CIB in Patient 1 was associated with selective deficits in executive functions and attention, additional visuospatial deficits were observed in Patients 2 and 3. The latter two patients showed a general visuoconstructional deficit. These case studies support a primary attentional account of CIB but also suggest that visuoconstructional impairments may contribute to the emergence of CIB, in some subjects. This evidence argues for different types of CIB with different cognitive and neural underpinnings. Furthermore, the data support the hypothesis of a differential involvement of fronto-parietal network in CIB.

## Introduction

Neuropsychological examination of constructional abilities encompasses copy drawing, drawing from memory, and three-dimensional constructions. When testing constructional abilities in patients suffering from different diseases (e.g., dementia, stroke, encephalitis, Parkinson disease, and corticobasal degeneration), clinicians occasionally observe a peculiar behavior in graphic copying, known as closing-in behavior (CIB) (Ambron and Della Sala, 2017). Patients with CIB place the copy abnormally close to the model (Near CIB) or overlap the copy with the model (Overlap CIB) (Ambron and Della Sala, 2017). This tendency is often associated with poor accuracy of the copy reproduction, leading to the interpretation of CIB as an aspect of constructional apraxia (Critchley, 1953).

However, CIB is not only observed in graphic copying, but it has also been noted in writing (Stengel, 1944; Suzuki et al., 2003) and gesture imitation (Kwon et al., 2002; McIntosh et al., 2008). In writing, CIB has been manifested as a tendency to superimpose writing upon previously written letters (Stengel, 1944), to anchor the writing to visible marks on the paper (Mayer Gross, 1935), or in copying kanji characters (Suzuki et al., 2003). Several authors have described CIB in gesture imitation (Mayer Gross, 1935; De Ajuriaguerra et al., 1949; Kwon et al., 2002; McIntosh et al., 2008). Kwon et al. (2002) described the case of a patient with corticobasal degeneration who exhibited severe ideomotor apraxia. When asked to imitate meaningless gestures presented by the examiner, this patient showed the tendency to approach, to touch and overlap his hand with the examiner’s hand. A similar tendency was noted also in a patient suffering from Alzheimer’s disease (AD) (McIntosh et al., 2008), who showed CIB in both graphic copying and imitation of gestures. In this patient, the presence of CIB co-occurred with the presence of both limb and constructional apraxia.

The cognitive and neuroanatomical bases of CIB are still a matter of debate. There are two major, competing interpretations of CIB (Ambron and Della Sala, 2017; Ambron et al., 2018a). The “compensation” hypothesis links CIB to visuoconstructional, visuospatial and working memory deficits, so that patients have difficulty in the visuospatial analysis of the model and/or in holding this representation in visual working memory. In contrast, the “attraction” account provides CIB an independent status from constructional deficits and considers CIB to be an extreme manifestation of a default tendency of the motor system, so that the actions would be performed toward the focus of attention. Further specification of this interpretation proposes that CIB would represent a primitive coupling between attention and action released by a decrease of attention and/or executive resources (McIntosh et al., 2008; Ambron et al., 2018a,b). The accounts make different predictions regarding the anatomic bases of CIB. As the compensation account postulates an association between CIB and visuo-contructional and working memory impairments, it predicts an association between CIB and involvement of posterior brain areas including the parietal lobe. On the contrary, the attraction account proposes that CIB is a consequence of a deficit in attention and/or executive resources and consequently implicates frontal lobe dysfunction (Kwon et al., 2002; Lepore et al., 2005).

Both accounts have received some support from single case and correlational studies. In single case descriptions, CIB has been reported in association with visuospatial and/or memory deficits, as well as with executive and attentional deficits (see Ambron and Della Sala, 2017 for a review). Most commonly, CIB has been reported in association with severe constructional deficits (Ambron and Della Sala, 2017), but it was noted with mild constructional deficits in patients suffering from corticobasal degeneration (Conson et al., 2009) and right fronto-temporal stroke (Conson et al., 2016).

Correlational studies have focused on identifying the difference in cognitive performance between patients with or without CIB or on specifying the best predictor of the phenomenon. Cohort studies in patients with AD (Ambron et al., 2009b; De Lucia et al., 2013) showed a preferential association between the presence of CIB and attentional/executive and visuo-contructional impairment; whereas visuospatial or memory deficits did not account for the presence of CIB in these samples. Similarly, a study exploring CIB in patients with mild cognitive impairment (MCI) showed that the phenomenon is more common in multidomain than amnestic MCI and that the decrease in executive functions (measured with the Frontal Assessment Battery) rather than memory or visuoconstructional abilities, distinguished between patients with and without CIB. These results were replicated in patients with Parkinson’s disease (De Lucia et al., 2015), in whom impairment in executive functions, but no other cognitive or motor impairments, predicted the presence of CIB. Finally, a cohort study (Ambron et al., 2009a) has shown that CIB is as common in AD as in FTD, but it presents different characteristics in these two populations. CIB in FTD is not influenced by the visuo-spatial demand of the copying task, whereas in AD the frequency of CIB increased with the complexity of the task, suggesting a possible additional role of visuospatial abilities in the appearance of CIB in this population. A different study in subjects with AD supported the compensation hypothesis (Serra et al., 2010). Indeed, Serra et al. (2010) found patients with CIB to be more impaired in visuospatial tasks than patients without CIB, while similar performance in executive tasks and frequency of frontal lobe associated-symptoms, were observed between the two groups.

Regarding the neuroanatomical bases of CIB, findings are also controversial. In single cases, CIB has been reported in patients suffering from damage to posterior parietal lobe (Critchley, 1953; Suzuki et al., 2003) and parietal-temporal areas (Kwon et al., 2002) as well as subjects with frontal (Lepore et al., 2005) or fronto-temporal regions (Conson et al., 2016). At the group level, studies have focused on inferring the neuroanatomical bases of CIB based on the frequency of CIB in different clinical groups. CIB is more common in patients suffering from dementia than in focal brain damage (Gainotti, 1972). CIB has been reported in AD (Ambron et al., 2009b; De Lucia et al., 2013, 2014), FTD, and vascular dementia (Ambron et al., 2009a; De Lucia et al., 2014; Grossi et al., 2015). The preferential association between CIB and dementia, and the observation that CIB is as frequent in AD as FTD when patients are matched for dementia severity (Ambron et al., 2009a), suggests that CIB may appear as a consequence of lesions in different brain areas (Ambron and Della Sala, 2017). A direct investigation of the neuroanatomical bases of CIB has been carried out only in one group study with patients with AD and matched controls (Kwon et al., 2015). Using, voxel-based morphometry (VBM), the results of this study showed that decrease of the distance between the model and copy was associated with a reduction of gray matter volume in the orbitofrontal cortex. Based on these data, one may suggest that CIB reflects disruption in the fronto-parietal network.

The investigation of CIB has benefited greatly from single case investigations. Our present study is in this tradition. We describe three patients who exhibit different forms of CIB. In one patient with stroke, CIB appeared independent of constructional and severe visuospatial and memory deficits; in two patients with dementia, in contrast, CIB was associated with profound deficits in visuo-constructional abilities, praxis and dressing. Imaging data showed abnormalities in parietal areas in all three patients, but further analyses in patients with dementia showed alterations in the connections between fronto-parietal regions. These cases are discussed with reference to the theoretical accounts of CIB and provide important evidence regarding the nature and anatomical bases of CIB.

## Materials and Methods

Three single cases were examined by different clinicians at different hospitals and therefore underwent different cognitive assessment. Imaging data were acquired for clinical purposes. Patient 1 underwent Computed Tomography (CT), while Patients 2 and 3 underwent structural magnetic resonance imaging (MRI) and functional MRI (fMRI). This study was approved by the Regional Ethics Committee of Friuli-Venezia Giulia (Italy) at the “Ente per la Gestione Accentrata dei Servizi Condivisi (E.G.A.S.)” (Udine, Italy) and participants signed an informed consent prior the cognitive and imaging testing.

### Procedure

Patients underwent different constructional tasks, but the same scoring criteria were applied to identify the presence of CIB and constructional deficits. Specifically, we scored the presence of Overlap CIB, when the copy touched or overlapped the model; Near CIB was identified when the copy was ≤10 mm from the model. Constructional abilities for each copied figure were rated using a score from 0 to 2, with 0 indicating poor accuracy of the copy (the copy was unrecognizable); 1 indicating moderate accuracy (the copy is partially recognizable, as some elementals are missing or spatially misplaced); 2 indicating good accuracy (the copy is recognizable and well reproduced).

### Cases Description

#### Patient 1

Patient 1 was a late-middle-aged patient who suffered from a hemorrhagic stroke in the right posterior parietal lobe. After 1 month from the accident, this patient was admitted to the Rehabilitation ward of Ospedale Riuniti (Trieste), where s/he underwent a neuropsychological evaluation and an intensive rehabilitation program for left hemiparesis.

##### General neuropsychological assessment

During the neuropsychological evaluation, Patient 1 showed temporal disorientation, as s/he was unable to provide the date and time of the evaluation, but preserved personal and spatial orientation.

Language abilities (both comprehension and production) were preserved, but the contents of his/her communication were often vague and confused. Cognitive examination confirmed this observation, as Patient 1 performed within the normal limits in the language tasks (see **Table 1**). Importantly, Patient 1 showed preserved visuospatial and working memory abilities (both verbal and visuospatial). S/he showed inconsistent performance when asked to recall items from long-term memory (i.e., his/her performance was below the normal range in one test and within the normal range in the other). The core cognitive alterations in Patient 1 were observed in attention and executive tasks. When tested for cognitive flexibility and the ability to change cognitive strategies depending on the task requirement (Weigl’s and Wisconsin Sorting card test), Patient 1 showed difficulty in disengaging attention from his/her current cognitive set to shift toward a new cognitive strategy. Significant impairment was also observed in the ability to shift attention across visual stimuli, as measured by the Trail Making Test. Patient 1 did not exhibit neglect on line bisection or letter cancellation tasks (see **Table 1**). S/he showed marked difficulties in exploring and identifying stimuli randomly distributed in space (Star Cancellation Test). S/he rescanned the same portion of space showing perseverations, but his/her performance was consistent across hemispaces, suggesting a difficulty disengaging and shifting attention to a new target. Personal neglect was clinically tested in the acute phase but not observed. It was not noted during the time of the neuropsychological assessment and neither during the period of rehabilitation.

**Table 1:** Patients’ raw (and adjusted) performance in the neuropsychological tasks.

	Patient 1	Patient 2	Patient 3	Cut off§
Addenbrooke’s cognitive examination (Pigliautile et al., 2011)		35^∗^	41^∗^	<88; 0–100
Mini Mental State Examination (Magni et al., 1996)		19^∗^	17^∗^	<24; 0–30
**Language**				
Picture naming	30			0–30
Picture naming (Laiacona et al., 2016) Living Not living		10^∗^ 7^∗^	19^∗^ 14^∗^	0–30; 0–30;
Verbal fluency (Carlesimo et al., 1996)	20 (24.7)	13 (20.7)	3 (11.6)^∗^	<17.35
Semantic fluency (Novelli et al., 1986)	32 (38)	7 (15)^∗^	5(15)^∗^	<24
Token test (De Renzi and Faglioni, 1978)		20 (20.5)^∗^	17 (17.75) ^∗^	29; 0–36
**Memory**				
***Working memory***				
Digit span (Monaco et al., 2013): forward	6 (6.49)	4 (4.39)	3 (3.51)^∗^	<4.26; 0–9
Backward	4 (4.52)	N.E.	2 (2.64)^∗^	<2.65; 0–9
Corsi span (Monaco et al., 2013): forward	5 (5.50)	2 (2.44)^∗^	2 (2.56)^∗^	<3.46; 0–9
Backward	3 (3.42)			<3.08; 0–9
***Memory***				
Prose memory (Novelli et al., 1986)	8.5 (11.5)			≤7.5; 0–28
Short story (Spinnler and Tognoni, 1987)	3.3 (3.55)^∗^	N.E.	6.5 (7)^∗^	<4.50; 1–16
Rey’s words (Carlesimo et al., 1996): immediate recall		10 (16.1)^∗^	N.E.	<28.5; 0–75
Rey’s words (Carlesimo et al., 1996): differed recall		1^∗^		≤4.6; 0–15
**Visuo-perceptual**				
Visual search task (Spinnler and Tognoni, 1987)	44 (45)	N.E.	12 (14)^∗^	<31; 0–60
Trail Making Test (Giovagnoli et al., 1996): Part A	97 (71)			>94
Minimal Feature Viewing Task (Riddoch and Humphreys, 1993)	25			0–25
Raven progressive matrices (Carlesimo et al., 1996)	34 (37.3)	N.E.	12 (16.5)^∗^	18.98; 0–36
Visual Object and Space Perception Battery (VOSP) (Warrington and James, 1991): screening		20	18	0–20
VOSP letters		0^∗^	11^∗^	0–20
VOSP Silhouettes living			5^∗^	0–15
VOSP Silhouettes not-living			4^∗^	0–15
**Attention and executive functions**				
Weigl’s Sorting Test (Spinnler and Tognoni, 1987)	4 (4.25)^∗^			
Modified Card Sorting Test (Caffarra et al., 2004): categories	1^∗^			<2; 0–6
Perseverations	8 (7.25)^∗^			<6.41
Trail Making Test (Giovagnoli et al., 1996): Part B	370 (282)^∗^	N.E.		>283
Frontal assessment battery (Appollonio et al., 2005)		6^∗^	6^∗^	<13.5; 0–18
Frontal Behavioral Inventory (Alberici et al., 2007)		15	4	>11.4; 0–23
Pyramid and palm tree test (Gamboz et al., 2009)		31^∗^		0–52
**Neglect**				
Behavioral Inattention Test (Wilson et al., 1987)				
Line bisection	9			≤7; 0–9
Star cancellation	51^∗^			≤51; 0–54
Letter cancellation	39			≤32; 0–40
Figure and shape copying	4			≤3; 0–4
Representational drawings	3			≤2; 0–3
**Apraxia**				
Ideomotor apraxia (Spinnler and Tognoni, 1987)		12^∗^		0–20 cut-off: ≤16
Ideational apraxia (De Renzi and Lucchelli, 1988)		4^∗^		0–10
Gesture imitation (De Renzi et al., 1980)		59^∗^	42^∗^	0–72
**Face recognition**				
Facial recognition test (Benton et al., 1983)		9 (25)^∗^		

*^§^ Cut offs are defined as above or below a certain score depending on the specific test. N.E., not executable because the patient does not follow the roles. ^∗^Performance below the normal range.*

##### Assessment of CIB

**FIGURE 1:**
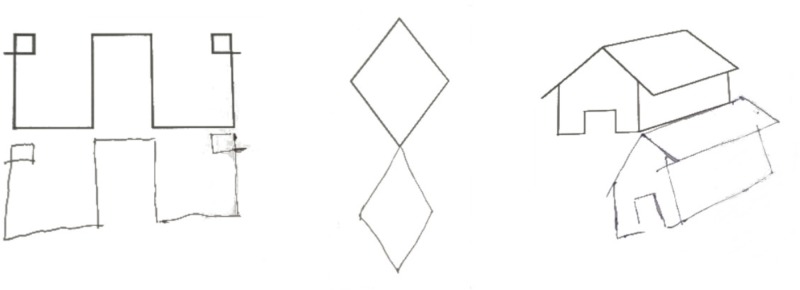
Representations of the graphic copying of Patient 1 showing CIB and good accuracy of the copy.

**FIGURE 2:**
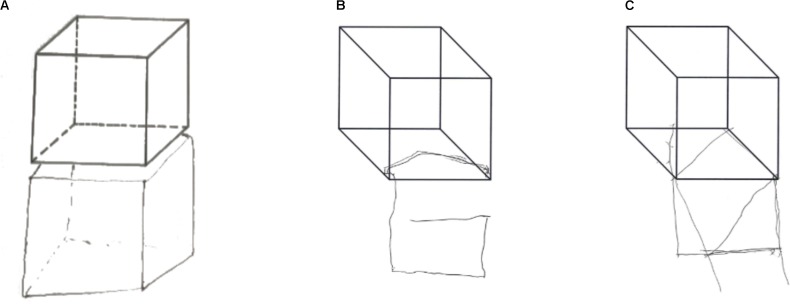
Patient 1 **(A)**, Patient 2 **(B)** and Patient 3 **(C)** graphic copying of the cube: all patients showed overlap CIB. Patient 1 shows a good accuracy of the copy, while Patient 2 and 3 shows CIB in association with constructional apraxia.

When tested for constructional apraxia, this patient was able to reproduce the model accurately, but his/her reproduction touched and overlapped the model to be copied. S/he was asked to copy the following visual stimuli: a square, a diamond, a star, a lateral extended geometrical shape, a complex triangular shape, a house, two cubes, and Luria’s figure. S/he was also asked to copy complex figures including one with a big rectangular triangle-triangle-diamond-small isosceles triangle and another containing a small square, big circle and big isosceles triangle. CIB was noted in seven copying tasks: Overlap CIB was observed in four tasks (cubes, house, diamond), while Near CIB was noted in the square, the Greek and in Luria’s figure. In line with previous observations of CIB in Luria’s figure (Luria, 1966), Patient 1’s line drawing veered progressively toward the model. Interestingly, Patient 1’s copy of the figures was accurate; there was no constructional apraxia (see **Figure 1**). In copying a house, this patient anchored his lines drawing to the original model, so that it appeared to be shifted toward the right side of the paper (see **Figure 1**). If this performance reminds of the one observed in patients’ with neglect, the presence of visuospatial neglect was not noted in neuropsychological assessment and could not account for Patient 1’s performance in this line drawing task, which rather reflected the presence of CIB. The only errors were an omission in the complex triangular shape, and the omission of an edge of a cube due to the presence of CIB (see **Figure 2**). Drawing from memory was also preserved as shown from the performance within the normal range in a clock drawing from memory task (score 8.5; test range 0–10, cut off < 3) (Mondini et al., 2003).

During motor rehabilitation, the physiotherapist noted a peculiar tendency of this patient to move toward attended stimuli. During upper limb rehabilitation, the therapist moved the paretic limb in a specific position on the table and asked the patient to move the right ipsilesional hand in a position that mirrored the contralesional hand. When blindfolded, Patient 1 was unable to do so and moved his/her hand toward the contralesional hand and positioned the ipsilesional hand very close to the contralesional hand (cf, McIntosh et al., 2008). To the best of our knowledge, this represents the first observation of CIB toward the patient’s own body part; gestural CIB was not assessed.

#### Patient 2

Patient 2 was a late-middle-aged patient who received a diagnosis of dementia 1 year before the assessment reported here. At the time of the assessment, this patient presented with delusional jealousy and repetitive formed visual hallucinations. S/he tended to get lost in familiar places and his/her partner reported episodes of misrecognition of his/her home. Patient 2’s daily life was highly compromised and s/he relied on the partner for most indoor and outdoor activities. On examination, Patient 2 showed good language skills, however, the structure of his/her communication was chaotic and hard to follow. S/he was cooperative, but with difficulty in sustaining attention. S/he presented masked facies, rapid mood changes, and profound anosognosia for his/her deficits.

##### General neuropsychological assessment

Neuropsychological examination revealed a global deterioration, as shown by low performance in Addenbrooke’s Cognitive Examination, the Mini Mental State Exam and other cognitive measures (see **Table 1**). Patient 2 did not show optic ataxia or neglect. Patient 2 was presented with 16 Navon stimuli comprised of small letters in the shape of a large letter. S/he was unable to name the global shape but named the local shape on 14/16 trials; she produced visual errors apparently involving the small letter on two trials.

Patient 2 showed profound constructional, limb and dressing apraxias. She was impaired in imitation of meaningless and meaningful gestures, and was unable to demonstrate the use of an object even when permitted to hold it. She was unable to dress herself; s/he was not able to put on gloves and showed additional difficulties in recognizing left and right gloves. She was unable to determine if she or the examiner were wearing clothes correctly. Although she could name doll’s clothing items (albeit imperfectly) and describe the manner in which a doll should be dressed, she was unable to dress the doll.

##### Assessment of CIB

**FIGURE 3:**
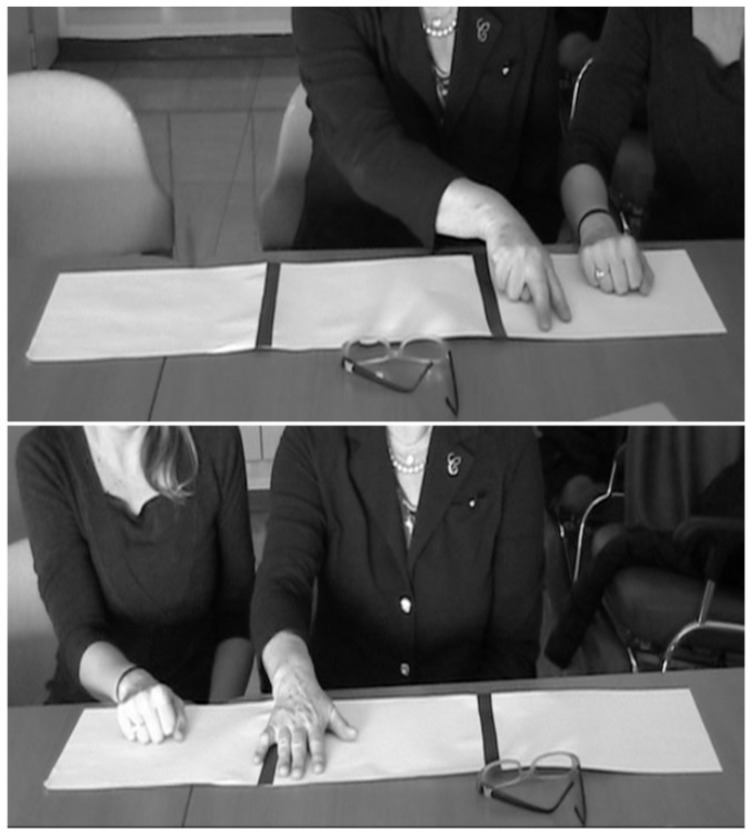
Example of Overlap **(Top)** and Near **(Bottom)** CIB observed in imitation of gestures in Patient 2.

Patient 2 was asked to copy 4 figures (square, two overlapped squares, a cube, and Luria’s figure); s/he showed poor accuracy in the copy of all the figures (2/8). CIB was not observed when copying the easier shapes but Overlap CIB emerged in copying the cube (see **Figure 1**). Gestural CIB was also tested asking Patient 2 to imitate the examiner gesture (fist or “V” hand shape) in a specific working space, while the examiner performed the gesture at the right or left side of the patient (McIntosh et al., 2008). Division lines delimited the patient’s working space and in two occasions Patient 2 performed the gesture on the division line, while in one s/he crossed the division line and performed the gesture in the examiner working space (near CIB) (see **Figure 3**). Drawing from memory was also impaired (1/5 in the clock drawing of the Addenbrooke’s Cognitive Examination).

#### Patient 3

Patient 3 was an elderly retired architect with a history of slowly progressive dementia.

##### General neuropsychological assessment

At the time of testing s/he had a moderate impairment, scoring 17/30 on the MMSE; s/he was able to take walks alone in her immediate neighborhood but required assistance with personal hygiene. On examination, s/he had marked difficulties in sustaining attention but was aware of her/his difficulties and showed frustration. S/he showed impaired performance in most cognitive tasks presented during the neuropsychological examination (see **Table 1**), suggesting that deficits encompassed multiple cognitive domains.

Patient 3 performed poorly (2/8) when asked to copy the same figures presented to Patient 2 (square, two overlapped squares, a cube, and Luria’s Figure). Near CIB was observed in copying the square and Luria’s Figure, while overlap CIB was observed in copying the overlapped squares and the cube (see **Figure 2**). The presence of constructional apraxia was also confirmed from the low score in immediate copy (score of 11/36) and delayed production (score of 3/36) of Rey’s Figure (Caffarra et al., 2002) and the clock drawing task of the Addenbrooke’s Cognitive Examination (score of 0/5). As Patient 2, Patient 3 was also tested for the presence of CIB in gesture imitation and although s/he was not able to copy gestures accurately but s/he did not show CIB.

##### Assessment of CIB

Patient 3’s neuropsychological examination revealed a similar cognitive profile as Patient 2, with a global cognitive impairment that affected visuospatial, memory, attention, and executive functions. In addition, the language impairment was profound, involving both production and comprehension. Like Patient 2, Patient 3 showed difficulties recognizing and naming different items of clothing (score of 21/43) and reported dressing apraxia. S/he did not show neglect but had difficulty reaching to non-foveated targets. When shown Navon figures, s/he named the local figure first but with additional prompting named the global figure on 10/16 trials.

### Brain Analyses

#### Lesion Mapping

Patient 1 underwent a CT scan in the acute stage 1 month before neuropsychological examination. For every slice, an expert neuroradiologist traced the Volume-of-Interest (VOI) using MRIcron^1^; the lesion and scan were then normalized to MNI space using statistical parametric mapping (SPM12) software^2^.

#### Structural and Functional Magnetic Resonance Imaging Analyses

For Patient 2 and Patient 3, both structural and resting fMRI data were collected. Structural T1-weighted images were acquired with the following parameters: flip angle = 12°, TR/TE = 8.1/3.7 ms, number of slices = 170, and voxel size = 1 mm × 1 mm × 1 mm. The imaging protocol also included the acquisition of an echo-planar imaging sequence with flip angle = 90°, TR/TE = 2500/35 ms, number of slices = 36, voxel size = 3.5 mm × 3.5 mm × 3 mm, number of volumes = 200. Imaging data were also collected for 20 controls (13 women; age *M* = 70.2; *SD* = 4.2; education *M* = 11.5; *SD* = 3.2) with a Mini Mental State Examination within the normal range (scores > 25), without any history of severe neurological and psychological disorders, and without CIB in the graphic copying tasks.

Structural MRI scans were pre-processed using the computational anatomy toolbox (cat12^3^) of SPM12. Images were segmented into gray matter, white matter and CSF, and subsequently were normalized to Montreal Neurological Institute (MNI) space. Finally, images were smoothed with a full-width half maximum (FWHM) kernel of 8 mm. Healthy controls pre-processed images were used to compute a mean and a standard deviation image, in order to transform patients’ images (Patients 2 and 3) into *z*-scores maps. *Z*-scores maps thresholds were set below −3.

Resting state fMRI was acquired with 3 Tesla MRI (Philips Medical Systems Achieva). fMRI scans were pre-processed and analyzed using conn^4^ (Whitfield-Gabrieli and Nieto-Castanon, 2012). Pre-processing included slice-time correction, realignment, normalization (MNI space) and smoothing with an 8 mm FWHM kernel. Moreover, images were de-noised (white matter, CSF, and movement parameters) and first-level connectivity analyses were performed correlating fluctuations over time in BOLD-related activity between specific atlas-based (Tzourio-Mazoyer et al., 2002) regions-of-interests (ROI). As we were specifically interested in parietal and frontal regions, we included 6 ROIs: left and right superior frontal gyri (SFG), anterior supramarginal gyri (SMG) and inferior lateral occipital cortices (LOC). To compare correlations in the activity patterns in resting state data for Patients 2 and 3 to control group data we used the method of Crawford and Garthwaite (2002) with one-tailed *p*-values.

## Results

### Lesion Mapping

Patient 1 exhibited a large (29,168 mm^3^) lesion in the right parietal lobe (lateral, superior, and inferior), which extended toward the paracentral and precentral gyrus.

### VBM and Resting State Analyses

The VBM analysis in Patient 2 demonstrated reduced gray matter volume in most the right fusiform and, inferior and middle temporal areas of the right hemisphere. Atrophy was also observed in the left inferior frontal, right parietal (both superior and inferior), and lateral occipital areas. Similar patterns of brain atrophy were also observed in Patient 3. In this patient, major GM volume reduction with respect to controls was observed in the right inferior temporal and in both right and left middle temporal gyri. GM changes encompassed superior parietal lobe, middle occipital and fusiform areas of the right hemisphere. Lesions extended also to right anterior cingulate cortex.

In Patients 2 and 3 we found reduced correlations between right superior frontal gyrus and the anterior portion of the right and left SMG, and right and left LOC (all *p*s < 0.05, except for Patient 2: correlation between right superior frontal gyrus and right LOC *p* = 0.059); the correlation between left and right SFG was not different than healthy controls (*p* > 0.05) (see **Tables 2**, **3**). Reductions in the correlation between left SFG and right LOC in Patient 2 (*p* < 0.05), and right (*p* < 0.01) and left (marginally significant: *p* = 0.07) SMG, and right LOC (marginally significant: *p* = 0.07) in Patient 3, and were also found. All the other comparisons did not reach the significance level (all *p*s > 0.05).

**Table 2:** Pattern of functional connectivity of Patient 2.

Right SFG	Right SMG	Right LOC	Left SFG	Left SMG	Left LOC	Patient 2
	−4.00^∗∗∗^	−1.68ˆ	0.75	−3.44^∗∗^	−3.07^∗∗^	Right SFG
		−0.76	−1.45	0.89	0.03	Right SMG
			−2.20^∗^	−1.02	−1.32	Right LOC
				−1.44	−1.44	Left SFG
					−0.25	Left SMG
						Left LOC

*The table reports the difference in the correlation between different ROIs, with respect to the healthy control group. ^∗^p < 0.05, ^∗∗^p < 0.01, ^∗∗∗^p < 0.001, ˆp = 0.059.*

**Table 3:** Pattern of functional connectivity of Patient 3.

Right SFG	Right SMG	Right LOC	Left SFG	Left SMG	Left LOC	Patient 3
	−3.06^∗∗^	−2.00^∗^	−0.10	−2.15^∗^	−2.37^∗^	Right SFG
		0.08	−3.06^∗∗^	−1.44	−0.05	Right SMG
			−1.62ˆ	−0.24	−0.13	Right LOC
				−1.83ˆ	−1.28	Left SFG
					0.18	Left SMG
						Left LOC

*The table reports the difference in the correlation between different ROIs, with respect to the healthy control group. ^∗^p < 0.05, ^∗∗^p < 0.01, ^∗∗∗^p < 0.001, ˆp = 0.07.*

### Comparison Across Patients

**FIGURE 4:**
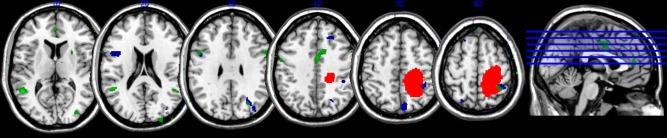
Maps of the damaged area of Patient 1 (red map) as result of lesion mapping, and of Patient 2 (blue map), and Patient 3 (green map) as result of VBM (slices 82, 92, 102, 112, 122, and 132). A common area of overlap is observed in the right posterior parietal lobe.

The lesions maps of the three patients with CIB are shown **Figure 4**. Areas of overlaps across the three patients can be noted in the right inferior and superior parietal cortex. In addition, minor points of lesions overlap were also observed in right supplementary motor and superior frontal areas. Additional similarities were observed in the GM reduction of Patient 2 and 3, effecting in particular the right inferior temporal area and the middle temporal gyrus bilaterally, left inferior frontal, and middle occipital area.

## Discussion

Three case studies of patients with CIB are presented: one suffering from stroke (Patient 1) and two from dementia (Patients 2 and 3). All three patients showed clear CIB (Overlap CIB) as their copies of figures often overlapped the model space. The patients differed in other, important respects, however. Patients 2 and 3 were unable to copy figures reliably, whereas Patient 1 performed adequately on this task. The association between CIB and constructional apraxia has often been observed, particularly when testing patients with dementia, leading some to suggest that CIB is a direct manifestation of constructional apraxia (Critchley, 1953; see also Ambron and Della Sala, 2017 for a review). This account, however, has been questioned in the past (McIntosh et al., 2008; Ambron et al., 2009b, 2012; Conson et al., 2009); data from Patient 1 further undermines the argument that CIB and constructional apraxia share a common basis and provides strong evidence that CIB can be observed as an independent phenomenon (see also Ambron et al., 2009b; Ambron and Della Sala, 2017).

The co-occurrence of CIB and constructional apraxia demonstrated by Patients 2 and 3 is common in patients with dementia, in whom the frequency and severity of both CIB and constructional deficits increases with the severity of dementia (Ambron et al., 2009a). As both Patients 2 and 3 were in moderate dementia stage, the co-occurrence of CIB and constructional apraxia in these patients is not surprising. Interestingly, in addition to constructional apraxia, both patients showed limb and dressing apraxia. These data suggest that these patients had a profound impairment of construction and action performance affecting both external and personal space.

Closing-in behavior was not observed only in copy drawing, but also in gesture imitation. When asked to imitate meaningless gesture, Patient 2 occasionally performed the gesture in the examiner’s action space. This observation supports and extends the single case description of a patient with dementia, who showed CIB in graphic copying and gesture imitation (McIntosh et al., 2008). Also, Patient 1 showed a peculiar form of CIB: when asked to move the ipsilesional hand in a position that mirrored contralesional hand, s/he moved the ipsilesional hand toward contralesional hand. This represents the first evidence of which we are aware that CIB can occur not only toward external stimuli, but also toward one’s own body. As praxis was not tested in this patient, we do not know whether gestural CIB observed in Patient 1 reflected a specific manifestation of CIB toward the patient’s own body or the appearance of the phenomenon across a wide range of gesture imitation tasks. On the other hand, alternative interpretations can be proposed to account for this peculiar behavior. Indeed, this patient’s brain lesion is compatible with the presence of personal neglect (Committeri et al., 2018) and body representational deficits (Schwoebel and Coslett, 2005). Personal neglect was not noted during the acute phase or during the neuropsychological assessment but its presence was not systematically tested. However, patients with personal neglect show a different behavior from the one observed in the present case study, in that they tend to neglect the contralesional side of the body, while Patient 1’s movements veered toward it. Furthermore, Patient 1 was not tested for alterations of body representation. It is possible that difficulties in identifying the position of his/her own body in space might have influenced the performance of the patients on this task which required proprioceptively guided reaching movements toward his/her own body.

In relation to the cognitive underpinning of CIB, all three patients showed alterations in attention and executive functions. For Patient 1 these represented the core cognitive alterations whereas for Patients 2 and 3 with moderate dementia all cognitive domains were affected. These data support the attraction account of CIB and the hypothesis that CIB may be caused by diminished attentional and executive capacities. More specifically, our data are consistent with the hypothesis that CIB is attributable to a difficulty in shifting attention from the model space and to a different action space (McIntosh et al., 2008; Ambron and Della Sala, 2017). Indeed, it is possible to speculate that CIB might reflect a specific difficulty in shifting attention from the specific elements of the figure. This interpretation would also account for the strong association between CIB and visuospatial deficits (Serra et al., 2010), as these deficits could reflect the secondary effect of a difficulty in shifting attention from the site of the model.

It should be noted that a number of aspects of the patients’ performance are consistent with those that of patients with the syndrome of “posterior cortical atrophy,” a disorder characterized by profound visuo-spatial deficits that is commonly observed in patients with Alzheimer Disease (Crutch et al., 2012). We have reported patients with this disorder, for example, who showed profound difficulties in identifying the global shape in Navon figures despite a preserved ability to recognize the local shape (Coslett et al., 1995). This behavior, as well as an inability to read or identify large words or objects with relatively preserved ability to recognize the same stimuli where presented in a smaller size (see also Saffran et al., 1990), was attributed to a reduction in the size of the “spotlight of visual attention.”

We suggest that impairment in the ability to shift attention, perhaps with an associated reduction in the domain to which attention can be allocated can account for many of the deficits exhibited by our patients. This account, for example, could explain the poor performance of these patients in the Trail Making or Star Cancellation tasks. This interpretation is also in line with the evidence that patients with CIB are more prone to distractor interference than patients without CIB, showing a marked deviation of the movement trajectory toward the focus of attention (Ambron et al., 2018b). This account further predicts that CIB would be more likely to appear as the size of the shape to be copied increases. Although the latter prediction has never been tested directly, the observation that CIB increases with the complexity of the figure to be copied seems to support this view (Ambron and Della Sala, 2017). Furthermore, extending the putative deficit in switching attention to other domains could explain several other aspects of the patients’ behavior. For example, the finding that gestures are copied in unusually close proximity to the examiner’s model is consistent with the hypothesis that attentional restriction affects both perceptual and action systems.

Finally, in this context, it should be noted that the proposal that CIB is related to an impairment in switching attention may also apply to Patient 1. Although we have no direct experimental evidence of such an impairment in this Patient, it is well known that parietal lobe lesions of the type exhibited by this Patient are associated with deficits in switching attention that may be profound (Posner et al., 1984; Hamilton et al., 2010).

In relation to the neural underpinning of CIB, the lesion overlaps showed a common region of damage in the right inferior and superior parietal lobe. This evidence supports previous reports of an association between CIB and parietal lobe lesions (Kwon et al., 2002; Suzuki et al., 2003) and the interpretation of CIB as a symptom of parietal damage (Critchley, 1953). Previous work has argued for an association between frontal brain damage and CIB (Kwon et al., 2002; Lepore et al., 2005; De Lucia et al., 2013). These findings might be reconciled by arguing that CIB is associated with alterations in the fronto-parietal network (Ambron and Della Sala, 2017). The fMRI data from Patients 2 and 3 support this interpretation. When compared with 20 controls, both patients showed a reduced correlation between the activity of the right superior frontal gyrus and right supramarginal gyrus as well as between the right superior frontal gyrus and right lateral occipital cortex.

The absence of constructional deficits in Patient 1, despite damage in parietal area, is also interesting for the discussion regarding the role of parietal lobe in the emergence of constructional apraxia (Gainotti and Trojano, 2018). Indeed, the classical interpretation of constructional apraxia as a specific symptom of parietal lobe damage has been recently revised: it has been proposed that the different streams connecting parietal with occipital and frontal lobes may have a prominent role in the different manifestations of constructional apraxia (Gainotti and Trojano, 2018).

To summarize, the single case descriptions provided in the present study inform the theoretical debate regarding the nature of CIB in several respects. First, the presence of CIB with good constructional skills reinforces the view of CIB as a disorder that may be independent of constructional apraxia. Second, the observation of CIB in imitation of gesture of own hand postures, suggests that this attraction toward the model to be reproduced can occur not only in external space, but also toward one’s own body. Third, the evidence that attentional deficits represent an impairment common to all the three patients, despite the difference across patients in CIB manifestations, supports the attraction hypothesis of CIB. However, this observation does not exclude an additional involvement of visuospatial and visuo-constructional deficits in the genesis of CIB in some patients (Serra et al., 2010). This evidence supports the hypothesis that CIB manifestations may across patients’ populations and may present different cognitive and neurological underpinnings (Ambron and Della Sala, 2017). On this line, imaging analyses point at the importance of parietal structures in the appearance of CIB, but further suggests that the connection between these areas and frontal and occipital region might be crucial in the appearance of CIB. It is possible that changes at distinctive levels of this network might induce different manifestation of CIB. Future studies should further investigate these interpretations.

A limitation of the present study is that as we described single cases observed in clinical practice the patients underwent different cognitive assessments and neuroimaging analyses. Most importantly, both graphic and gestural CIB were assessed using different tasks. For graphic CIB, patients underwent tasks of different complexity that, as previously discussed, might have enhanced the presence of CIB to different degrees. Gestural CIB was systematically investigated only in patients with dementia (Patients 2 and 3), while its presence was not tested in the patient with stroke (Patient 1). This represents an important limitation in the description of the latter case study. Indeed the peculiar behavior consisting in the tendency to move toward his/her own body during rehabilitation was not experimentally tested and definitive conclusions regarding the nature of this behavior cannot be drawn based on the present data. Furthermore, the use of different neuroimaging measures did not permit comparisons across all three patients. Although a common area of overlap in patients’ lesions was observed in parietal regions, definitive conclusion regarding the relationship between this area and CIB cannot be drawn. Indeed, lesions of patients with dementia were not limited to the parietal lobe but extended to a large variety of regions including fronto-occipital cortices. Furthermore, as functional connectivity data were not available for Patient 1, the involvement of the fronto-parietal network, beyond the damage of parietal areas, in the emergency of CIB cannot be totally excluded.

Despite these limitations, these single case descriptions represent a good example of how important and informative single case studies can be in particular when describing the peculiar and fascinating neuropsychological syndrome of CIB.

## Author Contributions

EA collected part of the data and drafted the manuscript. LP collected part of the data, analyzed the data, and revised the manuscript. AL collected part of the data and revised the manuscript. HC revised the manuscript.

## Conflict of Interest Statement

The authors declare that the research was conducted in the absence of any commercial or financial relationships that could be construed as a potential conflict of interest.

## Footnotes

1 http://people.cas.sc.edu/rorden/mricron/index.html

2 http://www.fil.ion.ucl.ac.uk/spm/software/spm12/

3 http://www.neuro.uni-jena.de/cat/

4 http://www.conn-toolbox.org

## References

[B1] Alberici A. Geroldi C. Cotelli M. Adorni A. Calabria M. Rossi G. (2007). The frontal behavioural inventory (Italian version) differentiates frontotemporal lobar degeneration variants from Alzheimer’s disease. *Neurol. Sci.* 28 80–86. 10.1007/s10072-007-0791-3 17464470

[B220] Ambron E. Allaria F. McIntosh R. D. Della Sala S. (2009a). Closing-in behaviour in fronto-temporal dementia. *J. Neurol.* 256 1004–1006. 10.1007/s00415-009-5027-4 19240954

[B2] Ambron E. Beschin N. Cerrone C. Della Sala S. (2018a). Closing-in behaviour: compensation or attraction. *Neuropsychology* 32 259–268. 10.1037/neu0000401 29049888

[B3] Ambron E. Cerrone C. Beschin N. Della Sala S. (2018b). Closing-in behavior and motor distractibility in persons with brain injury. *Arch. Clin. Neuropsychol.* 10.1093/arclin/acy033 [Epub ahead of print]. 10.1093/arclin/acy033 29688299

[B4] Ambron E. Della Sala S. (2017). A critical review of closing-in. *Neuropsychology* 31 105–117. 10.1037/neu0000295 27442452

[B6] Ambron E. Mcintosh R. D. Allaria F. Della Sala S. (2009b). A large-scale retrospective study of closing-in behaviour in Alzheimer’s disease. *J. Int. Neuropsychol. Soc.* 15 787–792. 10.1017/S1355617709990245 19570308

[B7] Ambron E. Della Sala S. McIntosh R. D. (2012). Closing-in behaviour and motor distractibility. *Neuropsychologia* 50 419–425. 10.1016/j.neuropsychologia.2011.12.019 22230227

[B8] Appollonio I. Leone M. Isella V. Piamarta F. Consoli T. Villa M. L. (2005). The frontal assessment battery (FAB): normative values in an Italian population sample. *Neurol. Sci.* 26 108–116. 10.1007/s10072-005-0443-4 15995827

[B9] Benton A. L. Sivan A. B. Hamsher K. Varney N. R. Spreen O. (1983). *Contribution to Neuropsychological Assessment.* New York, NY: Oxford University Press.

[B10] Caffarra P. Vezzadini G. Dieci F. Zonato F. Venneri A. (2002). Rey-Osterrieth complex figure: normative values in an Italian population sample. *Neurol. Sci.* 22 443–447. 10.1007/s100720200003 11976975

[B11] Caffarra P. Vezzadini G. Dieci F. Zonato F. Venneri A. (2004). Modified card sorting test: normative data. *J. Clin. Exp. Neuropsychol.* 26 246–250. 10.1076/jcen.26.2.246.28087 15202543

[B12] Carlesimo G. A. Caltagirone C. Gainotti G. (1996). The mental deterioration battery: normative data, diagnostic reliability and qualitative analyses of cognitive impairment. *Eur. Neurol.* 36 378–384. 10.1159/000117297 8954307

[B13] Committeri G. Piervincenzi C. Pizzamiglio L. (2018). Personal neglect: a comprehensive theoretical and anatomo–clinical review. *Neuropsychology.* 32 269–279. 10.1037/neu0000409 29620402

[B14] Conson M. Nuzzaci C. Sagliano L. Trojano L. (2016). Relationship between closing-in and spatial neglect: a case study. *Cogn. Behav. Neurol.* 29 44–50. 10.1097/WNN.0000000000000083 27008249

[B15] Conson M. Salzano S. Manzo V. Grossi D. Trojano L. (2009). Closing-in without severe drawing disorders: the “fatal” consequences of pathological attraction. *Cortex* 45 285–292. 10.1016/j.cortex.2007.11.013 18708188

[B16] Coslett H. B. Stark M. Rajaram S. Saffran E. M. (1995). Narrowing the spotlight: a visual attentional disorder in presumed Alzheimer’s disease. *Neurocase* 1 305–318. 10.1080/13554799508402375

[B17] Crawford J. R. Garthwaite P. H. (2002). Investigation of the single case in neuropsychology: confidence limits on the abnormality of test scores and test score differences. *Neuropsychologia* 40 1196–1208. 10.1016/S0028-3932(01)00224-X 11931923

[B18] Critchley M. (1953). “Constructional apraxia,” in *The Parietal Lobes*, ed. Arnold E. (London: Hafner), 172–202.

[B19] Crutch S. J. Lehmann M. Schott J. M. Rabinovici G. D. Rossor M. N. Fox N. C. (2012). Posterior cortical atrophy. *Lancet Neurol.* 11 170–178. 10.1016/S1474-4422(11)70289-7 22265212 PMC3740271

[B20] De Ajuriaguerra J. Zazzo R. Granjon N. (1949). Le phenomene d’accolement au model (closing-in) dans une syndrome d’apraxie oxycarbonn.e (Phenomenon of model pairing -closing-in in apraxia caused by carbon monoxide intoxication). *Encephale* 38 1–20.

[B21] De Lucia N. Grossi D. Fasanaro A. M. Carpi S. Trojano L. (2013). Frontal defects contribute to the genesis of closing-in in Alzheimer’s disease patients. *J. Int. Neuropsychol. Soc.* 19 802–808. 10.1017/S1355617713000568 23701672

[B22] De Lucia N. Grossi D. Trojano L. (2014). The genesis of closing-in in Alzheimer disease and vascular dementia: a comparative clinical and experimental study. *Neuropsychology* 28 312–318. 10.1037/neu0000036 24219604

[B23] De Lucia N. Trojano L. Vitale C. Grossi D. Barone P. Santangelo G. (2015). The closing-in phenomenon in Parkinson’s disease. *Parkinsonism Relat. Disord.* 21 793–796. 10.1016/j.parkreldis.2015.04.013 25936264

[B24] De Renzi E. Faglioni P. (1978). Normative data and screening power of a shortened version of the Token Test. *Cortex* 14 41–49. 10.1016/S0010-9452(78)80006-9 16295108

[B25] De Renzi E. Lucchelli F. (1988). Ideational apraxia. *Brain* 111 1173–1188. 10.1093/brain/111.5.1173 3179688

[B26] De Renzi E. Motti F. Nichelli P. (1980). Imitating gestures. A quantitative approach to ideomotor apraxia. *Arch. Neurol.* 37 6–10. 10.1001/archneur.1980.00500500036003 7350907

[B27] Gainotti G. (1972). A qualitative study of the “closing-in” symptom in normal children and in brain-damaged patients. *Neuropsychologia* 10 429–436. 10.1016/0028-3932(72)90005-X 4657525

[B28] Gainotti G. Trojano L. (2018). Constructional apraxia. *Handb. Clin. Neurol.* 151 331–348. 10.1016/B978-0-444-63622-5.00016-4 29519467

[B29] Gamboz N. Coluccia E. Iavarone A. Brandimonte M. A. (2009). Normative data for the pyramids and palm trees test in the elderly Italian population. *Neurol. Sci.* 30 453–458. 10.1007/s10072-009-0130-y 19768374

[B30] Giovagnoli A. R. Pesce M. Del Mascheroni S. Simoncelli M. Laiacona M. Capitani E. (1996). Trail making test: normative values from 287 normal adult controls. *Ital. J. Neurol. Sci.* 17 305–309. 10.1007/BF01997792 8915764

[B31] Grossi D. De Lucia N. Milan G. Trojano L. (2015). Relationships between environmental dependency and closing-in in patients with fronto-temporal dementia. *J. Int. Neuropsychol. Soc.* 21 1–7. 10.1017/S135561771400099X 25399546

[B32] Hamilton R. H. Stark M. Coslett H. B. (2010). Increased effect of target eccentricity on covert shifts of visual attention in patients with neglect. *Cortex* 46 68–76. 10.1016/j.cortex.2009.02.005 19345939 PMC2852117

[B33] Kwon S. Y. Lee E. S. Hong Y. J. Lim S. Ahn K. J. Yoon B. (2015). Anatomical correlates of the “closing-in” phenomenon. *Dement. Neurocogn. Dis.* 14 17–23. 10.12779/dnd.2015.14.1.17

[B34] Kwon J. C. Kang S. J. Lee B. H. Chin J. Heilman K. M. Na D. L. (2002). Manual approach during hand gesture imitation. *Arch. Neurol.* 59 1468–1475. 10.1001/archneur.59.9.1468 12223035

[B35] Laiacona M. Barbarotto R. Baratelli E. Capitani E. (2016). Revised and extended norms for a picture naming test sensitive to category dissociations. *Neurol. Sci.* 37 1499–1510. 10.1007/s10072-016-2611-0 27215621

[B36] Lepore M. Conson M. Grossi D. Trojano L. (2005). Multidirectional transpositions suggesting pathologic approach behavior after frontal stroke. *Neurology* 64 1615–1617. 10.1212/01.WNL.0000160398.27467.C0 15883326

[B37] Luria A. (1966). *Human Brain and Psychological Process.* New York, NY: Harper and Row.

[B38] Magni E. Binetti G. Bianchetti A. Rozzini R. Trabucchi M. (1996). Mini-mental state examination: a normative study in Italian elderly population. *Eur. J. Neurol.* 3 198–202. 10.1111/j.1468-1331.1996.tb00423.x 21284770

[B39] Mayer Gross W. (1935). Some observations on apraxia. *Proc. R. Soc. Med.* 28 63–72. 10.1177/003591573502800938 PMC2205720 19990372

[B40] McIntosh R. D. Ambron E. Della Sala S. (2008). Evidence for an attraction account of closing-in behaviour. *Cogn. Neuropsychol.* 25 376–394. 10.1080/02643290802028981 18587701

[B41] Monaco M. Costa A. Caltagirone C. Carlesimo G. A. (2013). Forward and backward span for verbal and visuo-spatial data: standardization and normative data from an Italian adult population. *Neurol. Sci.* 34 749–754. 10.1007/s10072-012-1130-x 22689311

[B42] Novelli G. Papagno C. Capitani E. Laiacona M. Vallar G. Cappa S. F. (1986). Tre test clinici di memoria verbale a lungo termine. *Arch. Psicol. Neurol. Psichiatr.* 47 278–296.

[B43] Pigliautile M. Ricci M. Mioshi E. Ercolani S. Mangialasche F. Monastero R. (2011). Validation study of the Italian Addenbrooke’s Cognitive Examination Revised in a young-old and old-old population. *Dement. Geriatr. Cogn. Disord.* 32 301–307. 10.1159/000334657 22262124

[B44] Posner M. I. Walker J. A. Friedrich F. J. Rafal R. D. (1984). Effects of parietal injury on covert orienting of attention. *J. Neurosci.* 4 1863–1874. 10.1523/JNEUROSCI.04-07-01863.1984 6737043 PMC6564871

[B45] Riddoch M. J. Humphreys G. W. (1993). *BORB, Birmingham Object Recognition Battery.* Hove: Psychology Press.

[B46] Saffran E. M. Fitzpatrick-DeSalme E. J. Coslett H. B. (1990). “Visual disturbances in dementia,” in *Issues in the Biology of Language and Cognition Modular Deficits in Alzheimer-Type Dementia* Vol. 44 ed. Schwartz M. F. (Cambridge, MA: The MIT Press), 297–327.

[B47] Schwoebel J. Coslett H. B. (2005). Evidence for multiple, distinct representations of the human body. *J. Cogn. Neurosci.* 17 543–553. 10.1162/0898929053467587 15829076

[B48] Serra L. Fadda L. Perri R. Caltagirone C. Carlesimo G. A. (2010). The closing-in phenomenon in the drawing performance of Alzheimer’s disease patients: a compensation account. *Cortex* 46 1031–1036. 10.1016/j.cortex.2009.08.010 19782972

[B49] Spinnler H. Tognoni G. (1987). Standardizzazione e taratura italiana di test neuropsicologici. *Ital. J. Neurol. Sci.* 8 8–20. 3330072

[B50] Stengel E. (1944). Loss of spatial orientation, constructional apraxia and Gerstmann’s syndrome. *J. Ment. Sci.* 90 753–760. 10.1192/bjp.90.380.753

[B51] Suzuki K. Otsuka Y. Endo K. Ejima A. Saito H. Fujii T. (2003). Visuospatial deficits due to impaired visual attention: investigation of two cases of slowly progressive visuospatial impairment. *Cortex* 39 327–341. 10.1016/S0010-9452(08)70112-6 12784892

[B52] Tzourio-Mazoyer N. Landeau B. Papathanassiou D. Crivello F. Etard O. Delcroix N. (2002). Automated anatomical labeling of activations in SPM using a macroscopic anatomical parcellation of the MNI MRI single-subject brain. *Neuroimage* 15 273–289. 10.1006/nimg.2001.0978 11771995

[B53] Warrington E. K. James M. (1991). *The Visual Object and Space Perception Battery.* Bury St Edmunds: Thames Valley Test Company.

[B54] Whitfield-Gabrieli S. Nieto-Castanon A. (2012). *Conn*: a functional connectivity toolbox for correlated and anticorrelated brain networks. *Brain Connect.* 2 125–141. 10.1089/brain.2012.0073 22642651

[B55] Wilson B. Cockburn J. Halligan P. W. (1987). *The Behavioural Inattention Test.* Bury St Edmunds: Thames Valley Test Company.

